# Three new remarkable species of the genus *Endonura* Cassagnau, 1979 from the Middle East and Central Asia (Collembola, Neanuridae, Neanurinae, Neanurini)

**DOI:** 10.3897/zookeys.673.12084

**Published:** 2017-05-15

**Authors:** Adrian Smolis, Masoumeh Shayanmehr, Nataliya Kuznetsova, Elham Yoosefi Lafooraki

**Affiliations:** 1 Institute of Environmental Biology, Department of Invertebrate Biology, Evolution and Conservation, University of Wrocław, Przybyszewskiego 65, 51-148 Wrocław, Poland; 2 Department of Plant Protection, Sari Agricultural Science and Nature Resources University, Sari, Mazandaran 578, Iran; 3 Institute of Biology and Chemistry, Moscow State Pedagogical University, Moscow 129164, Russia

**Keywords:** Feeding habits, Iran, labral modifications, springtails, taxonomy, Turkmenistan

## Abstract

New species belonging to the genus *Endonura* is described and illustrated in detail. *Endonura longirostris* **sp. n.**, from northern Iran, is distinctive due to an exceptionally elongate buccal cone compared to that of most other species of the genus. Other characteristic features of the species are the white body with pigmented eyes, a reduced chaetotaxy of the lateral part of the head, the thorax II–III and abdomen I–III with free chaetae De2 and 3, and abdomen IV with particularly short chaetae Di1. The main characteristics of *Endonura paracentaurea* **sp. n.** include a white body with dark pigmented eyes, a nonogival labrum, the presence of tubercles Di on the first thoracic segment and of microchaetae on the rudimentary furca. *Endonura turkmenica* **sp. n.** can be recognized by its bluish-grey body, labral chaetotaxy, free chaetae E on the head, and notably short chaetae De3 on the thorax and the abdomen. Short remarks on the possible importance of labral modifications are also provided.

## Introduction

Amongst springtails, primitive and wingless insects, the subfamily Neanurinae is one of the most diversified, widespread, and species-rich systematic units and evolutionary lineages. Up to date, Neanurinae encompasses nearly 800 species, classified into six tribes (Cassagnau 1989). The Neanurini is the second largest tribe within the subfamily, after Paleonurini, currently containing over 170 valid species belonging to 22 genera (Cassagnau 1989, Hopkin 1997, Deharveng and Bedos 2000, Deharveng et al. 2007, Smolis 2007, 2011, Mayvan et al. 2015). The genus considered in this paper, *Endonura* Cassagnau, 1979, is among the most thoroughly studied and largest genera within the tribe, with 48 valid species at the moment (Dallai 1983, Deharveng 1979, 1982, Fanciulli and Dallai 2008, Pozo and Simón 1981, Smolis and Kaprus’ 2003, 2009, Smolis 2008, 2016, Smolis et al. 2007, 2011, 2016a, b). Regarding the actual distribution of its members, *Endonura* is a Holarctic genus, with the highest concentration of forms in Europe. Recent discoveries, however, slowly change this picture as a notable number of newly described species has been recorded in the Middle East, the Caucasus, and Central Asia (Smolis et al. 2011, 2016a, b).

In 2008, the first author redefined *Endonura* as follows (Smolis 2008): 0–2 ocelli on either side of the head, reduced mouth parts usually with a thin mandible and a styliform maxilla, separate tubercles Di and De on the head, the non-cross-type of chaetotaxy on the head, and three or two tubercles on the penultimate abdominal segment. Amongst Neanurini, the genus is more similar, and presumably seems to be closely related, to two genera: *Cryptonura* Cassagnau, 1979, and *Deutonura* Cassagnau, 1979, which share the following characters with *Endonura*: maximum 2+2 eyes on the head, the non-cross-type of chaetotaxy, and differentiation in size of dorsal chaetae. Nonetheless, *Endonura* can be separated from the former by having two or three tubercles on the dorsal side of the penultimate abdominal segment (in *Cryptonura*, four tubercles), and from the latter by the separation of tubercles Di on the head (in *Deutonura*, fused).

The examination of rich materials of Neanurinae from northern Iran and Turkmenistan has revealed three unknown species of the genus. One of them seems to be particularly remarkable because of an exceptionally elongated and pointed labrum. Descriptions of all the three taxa are provided with comments on their affinities and the significance of labral modifications in the taxonomy and ecology of the genus and the subfamily Neanurinae.

## Material and methods

### Terminology

Terminology and layout of the tables used in the paper follow Deharveng (1983), Deharveng and Weiner (1984), Smolis and Deharveng (2006) and Smolis (2008).

### Abbreviations used

General morphology:

**Abd** abdomen,

**Ant** antenna,

**AOIII** sensory organ of antennal segment III,

**Cx** coxa,

**Fe** femur,

**Scx2** subcoxa 2,

**T** tibiotarsus,

**Th** thorax,

**Tr** trochanter,

**VT** ventral tube.

Groups of chaetae:

**Ag** antegenital,

**An** chaetae of anal lobes,

**ap** apical,

**ca** centroapical,

**cm** centromedial,

**cp** centroposterior,

**d** dorsal,

**Fu** furcal,

**vc** ventrocentral,

**Ve** or **ve** ventroexternal,

**Vea** ventroexternoanterior,

**Vem** ventroexternomedial,

**Vep** ventroexteroposterior,

**Vel** ventroexternolateral,

**Vec** ventroexternocentral,

**Vei** ventroexternointernal,

**Vi** or **vi** ventrointernal,

**Vl** ventrolateral.

Tubercles:

**Af** antenno–frontal,

**Cl** clypeal,

**De** dorsoexternal,

**Di** dorsointernal,

**Dl** dorsolateral,

**L** lateral,

**Oc** ocular,

**So** subocular.

Types of chaetae:

**A**, **B**, **C**, **D**, **E**, **O**, **So**, **L**, **Dl** cephalic chaetae,

**Ml** long macrochaeta,

**Mc** short macrochaeta,

**Mcc** very short macrochaeta,

**me** mesochaeta,

**mi** microchaeta,

**ms** s–microchaeta,

**S** or **s** chaeta s,

**bs** s–chaeta on Ant IV,

**miA** microchaetae on Ant IV,

**iv** ordinary chaetae on ventral Ant IV,

**or** organite of Ant IV,

**brs** border s–chaeta on Ant IV,

**i** ordinary chaeta on Ant IV,

**mou** cylindrical s–chaetae on Ant IV („soies mousses”),

**x** labial papilla x,

**L’** ordinary lateral chaeta on Abd V,

**B4**, **B5** ordinary chaetae on tibiotarsi.

The specimens were cleared in Nesbitt’s fluid, subsequently mounted on slides in Swan’s medium and studied using a Nikon Eclipse E600 phase contrast microscope. Figures were drawn with camera lucida and prepared for publication using Adobe Photoshop CS3.

Institutions of depository of materials: **DIBEC** Department of Invertebrate Biology, Evolution and Conservation, Institute of Environmental Biology, University of Wrocław, Poland and **MSPU** Moscow State Pedagogical University, Moscow, Russia.

## Taxonomy

### Endonura longirostris sp. n.

Taxon classificationAnimaliaCollembolaNeanuridae

http://zoobank.org/4255D53B-46C3-4428-BE7A-894D98925C26

Figs 1–10 , Table 1

#### Type material.

Holotype: adult female on slide, Iran, Mazandarn province, Behshahr region, Abbas-Abad forest (36°40'N; 53°32'E), leaf litter and soil, 28.III.2013, leg. E. Yoosefi Lafooraki (DIBEC). Paratypes: 2 females on slide, same data as holotype.

**Figures 1–10.**
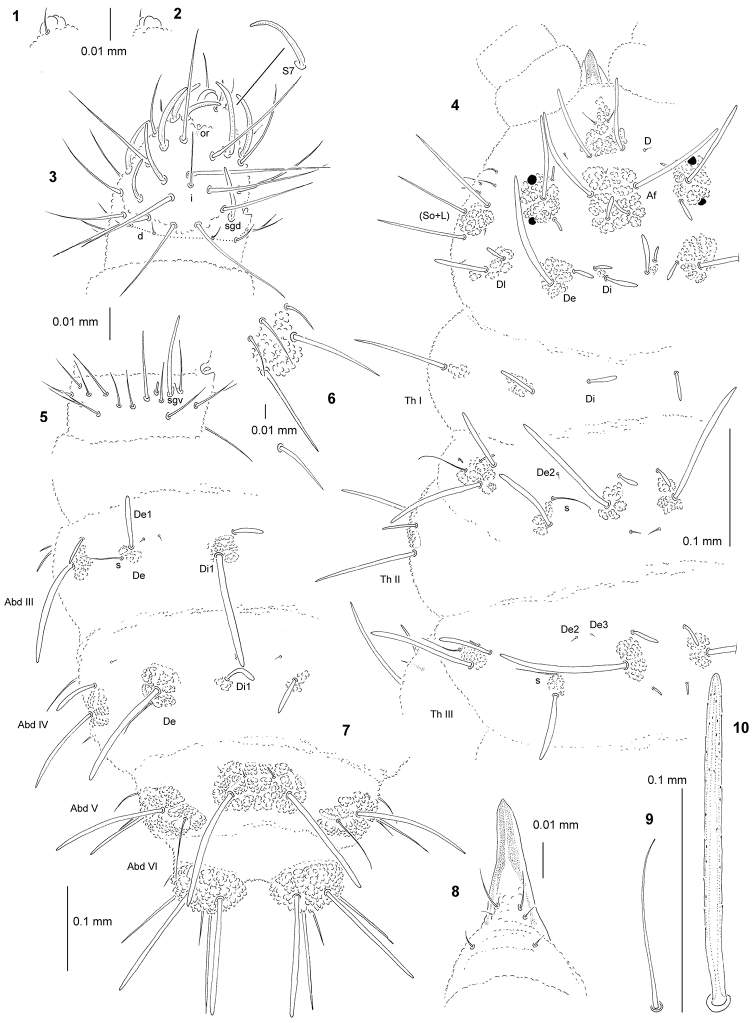
*Endonura longirostris* sp. n.: **1** apical bulb, dorsal view **2** apical bulb, ventral view **3** dorsal chaetotaxy of Ant III–IV **4** chaetotaxy of head and Th (holotype), dorsolateral view **5** ventral chaetotaxy of Ant III **6** tubercle L of Abd IV **7** dorsal chaetotaxy of Abd III–VI **8** chaetotaxy and ventral sclerifications of labrum **9** sensillum of Abd V **10** chaeta Di1 of Abd V.

**Table 1a.** Chaetotaxy of *Endonura longirostris* sp. n.: cephalic chaetotaxy dorsal side.

Tubercle	Number of chaetae	Types of chaetae	Names of chaetae
Cl	4	Ml me	F G
Af	8	Ml Mc Mcc or mi mi	B A C D
Oc	3	Ml Mc Mcc	Ocm Ocp Oca
Di	2	Ml Mcc	Di1 Di2
De	2	Mc Mcc	De1 De2
Dl	4	Ml Mc or Mcc mi	Dl5 Dl1 Dl2, Dl4
(L+So)	8	Ml mi me	L1, L4, So1 L2 So3–6

**Table 1b.** Chaetotaxy of *Endonura longirostris* sp. n.: chaetotaxy of antennae.

Segment, Group	Number of chaetae	Segment, Group	Number of chaetae adult
I	7	IV ap	or, 8 S, i, 12 mou, 6 brs, 2 iv
II	11		
III	5 sensilla AO III		
ve	5		8 bs, 5 miA
vc	4	ca	2 bs, 3 miA
vi	4	cm	3 bs, 1 miA
d	5	cp	8 miA, 1 brs

**Table 1c.** Chaetotaxy of *Endonura longirostris* sp. n.: postcephalic chaetotaxy.

	Terga				Legs				
	Di	De	Dl	L	Scx2	Cx	Tr	Fe	T
Th I	1	2	1	-	0	3	6	13	19
Th II	3	2+s	3+s+ms	3	2	7	6	12	19
Th III	3	3+s	3+s	3	2	8	6	11	18
							Sterna		
Abd I	2	3+s	2	3	VT: 4				
Abd II	2	3+s	2	3	Ve: 5; chaeta Ve1 present				
Abd III	2	3+s	2	3	Vel:4; Fu: 4–5 me, 0 mi				
Abd IV	2	2+s	3	5–6	Vel: 4; Vec: 2; Vei: 2; Vl: 4				
Abd V	(3+3)	7+s			Ag: 3; Vl: 1, L’: 1				
Abd VI		7			Ve: 14; An: 2mi				

#### Other material.

Juvenile on slide, Iran, Mazandarn province, Noor region, Kadirsar village (36°26'N; 51°49'E), leaf litter and soil, 1.III.2013, leg. E. Yoosefi Lafooraki (DIBEC).

#### Etymology.

The name *longirostris* refers to an exceptionally long buccal cone of this species.

#### Diagnosis.

Habitus typical of the genus *Endonura*. Dorsal tubercles present and well developed. 2+2 pigmented eyes. Buccal cone exceptionally long, labrum ogival. Head with chaetae A, B, C and D. Chaetae O and E absent. Tubercles Cl and Af separate. Tubercles Dl and (L+So) on head with 4 and 8 chaetae respectively. Tubercles Di on Th I absent. Tubercles De on Th II and III with 3 and 4 chaetae respectively. Tubercles L on Abd III and IV with 3 and 5-6 chaetae respectively. Abd IV and V with 8 and 3 tubercles respectively. Claw without inner tooth. Tibiotarsi with chaetae B4 and B5 short.

#### Description.

General. Body length (without antennae): 0.50 (juvenile) to 1.45 mm (holotype: 1.45 mm). Colour of the body white. 2+2 medium black eyes, in a typical arrangement for the genus (one anterior and one posterior eyes, Fig. 4).

*Chaetal morphology*. Dorsal ordinary chaetae of five types: long macrochaetae, short macrochaetae, very short macrochaetae, mesochaetae and microchaetae. Long macrochaetae thick, slightly arc-like or straight, narrowly sheathed, feebly serrated, apically rounded (Fig. 10). Macrochaetae Mc and Mcc morphologically similar to long macrochaetae, but much shorter. Mesochaetae similar to ventral chaetae, thin, smooth and pointed. Microchaetae similar to mesochaetae, but apparently short. S–chaetae of tergites thin, smooth and short, shorter than nearby macrochaetae (Figs 4, 7, 9).

*Antennae*. Typical of the genus. Dorsal chaetotaxy of Ant III–IV as in Fig. 3 and Table 1b. S–chaetae of Ant IV of medium length and moderately thickened, S2, S7 and S8 notably longer than others (Fig. 3). Apical vesicle distinct, trilobed (Figs 1, 2). Ventral chaetotaxy of Ant III–IV as in Fig. 5 and Table 1b.

*Mouthparts*. Buccal cone particularly long with labral sclerifications ogival. Labrum chaetotaxy: 0/2, 4 (Fig. 8). Labium with four basal, three distal and four lateral chaetae, papillae x absent. Maxilla styliform, mandible thin and tridentate.

*Dorsal chaetotaxy and tubercles*. Chaetotaxy of head reduced, chaetae E, O, Dl3, Dl6, So2 and L3 absent (Fig. 4). Tubercles Di on Th I not differentiated (Fig. 4). Thorax and abdomen with chaetae De2 and De3 free (Figs 4, 7). On Abd I–III, the line of chaetae De1–chaeta s non-perpendicular to the dorsomedian line. On Abd IV chaetae Di1 notably short (Fig. 7). Cryptopygy absent, Abd VI well visible from above.

*Ventral chaetotaxy*. On head, groups Vea, Vem and Vep with 3, 3, 3-4 chaetae respectively. Group Vi on head with 6 chaetae. On Abd IV, furca rudimentary without microchaetae. On Abd IV, tuberle L with one chaeta free (Fig. 6). On Abd V, chaetae Vl and L’ present.

*Legs*. Chaetotaxy of legs as in Table 1c. Claw without internal tooth. On tibiotarsi, chaeta M present and chaetae B4 and B5 relatively short and pointed.

#### Remarks.

Because of the presence of particularly elongated mouthparts and substantial reduction of chaetotaxy on lateral part of head, *E. longirostris* sp. n. strongly resembles *E. cretensis* (Ellis, 1976), known to date from Greece (Crete, Ellis 1976) and Israel (Smolis and Kaprus’ 2009). Nevertheless, these species can be easily distinguished from each other by the set of characters: body colour (in *longirostris* white, in *cretensis* bluish), presence/absence of chaeta O on head (in *longirostris* absent, in *cretensis* present), number of labial chaetae (in *longirostris* ten, in *cretensis* eight), presence/absence of tubercles Di on Th I (in *longirostris* absent, in *cretensis* present), number of ordinary chaetae De on Abd I-III (in *longirostris* three, in *cretensis* two) and number of chaetae Di on penultimate abdominal segment (in *longirostris* 3+3, in *cretensis* 1-2+1-2).

Viewing the recently published key to *Endonura* species (Smolis et al. 2016b), *E. longirostris* sp. n. is placed the nearest to *E. saleri* Fanciulli et Dallai, 2008, described from northeastern Italy (Fanciulli and Dallai 2008). However, these species differ in a number of details, including: shape of labrum (in *longirostris* ogival, in *saleri* nonogival), number of chaetae (L+So) on head (in *longirostris* eight, in *saleri* nine), number of chaetae Dl on head (in *longirostris* four, in *saleri* five), location of chaetae De 2 on Th II-III (in *longirostris* free, in *saleri* connected with tubercle De), location of chaetae De 2 on Abd I-III (in *longirostris* free, in *saleri* connected with tubercle De) and length of chaeta Di1 on Abd IV (in *longirostris* distinctly shorter than chaeta Di1 of Abd V, in *saleri* slightly shorter than chaeta Di1 of Abd V).

### Endonura paracentaurea sp. n.

Taxon classificationAnimaliaCollembolaNeanuridae

http://zoobank.org/9CC758B3-42F6-44D3-841B-D46B371997DD

Figs 11–19 , Table 2a

#### Type material.

Holotype: adult female on slide, Iran, Mazandarn province, Behshahr region, Abbas-Abad forest (36°40'N; 53°32'E), rooting wood, 28.III.2013, leg. E. Yoosefi Lafooraki (DIBEC). Paratypes: 3 females on slide, same data as holotype.

**Figures 11–19.**
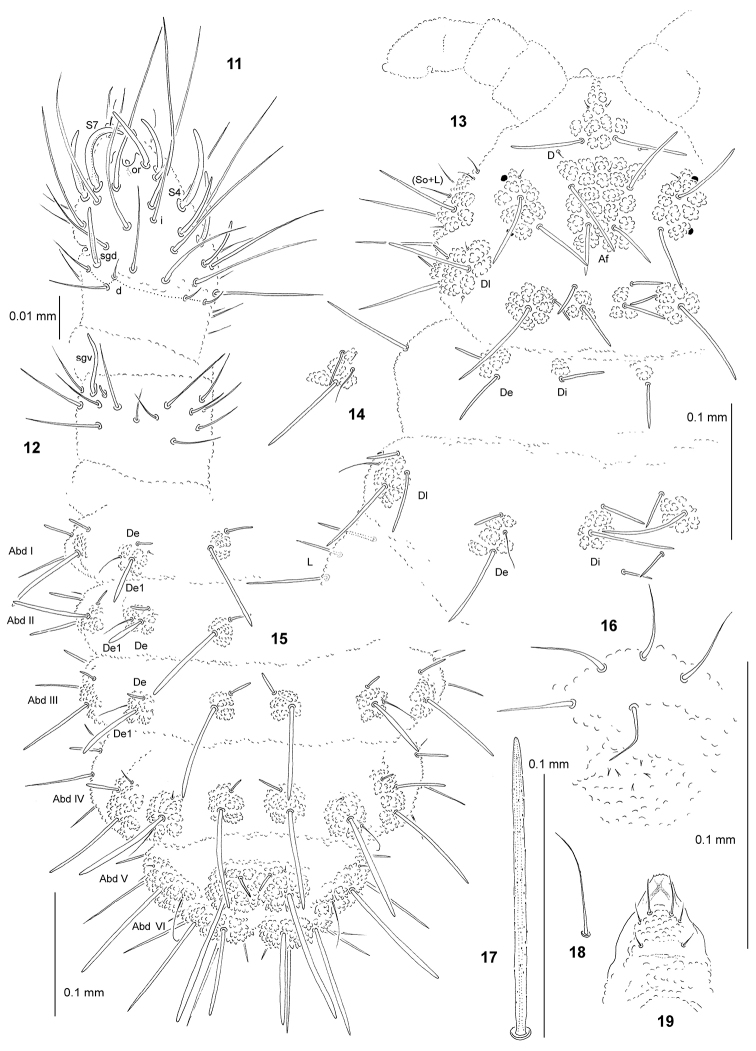
*Endonura paracentaurea* sp. n.: **11** dorsal chaetotaxy of Ant III–IV **12** ventral chaetotaxy of Ant III **13** chaetotaxy of head and Th I–II (holotype), dorsolateral view **14** chaetotaxy of tubercle De of Th III **15** dorsal chaetotaxy of Abd I–VI **16** furca rudimentary **17** chaeta Di1 of Abd V **18** sensillum of Abd V **19** chaetotaxy and ventral sclerifications of labrum.

**Table 2a.** Chaetotaxy of *Endonura paracentaurea* sp. n.: cephalic chaetotaxy dorsal side.

**Tubercle**	**Number of chaetae**	**Types of chaetae**	**Names of chaetae**
Cl	4	Ml me	F G
Af	8	Ml Mc mi	B A C, D
Oc	3	Ml Mc mi	Ocm Ocp Oca
Di	2	Ml Mc	Di1 Di2
De	2	Mc Mcc	De1 De2
Dl	6	Ml Mcc mi	Dl5, Dl1 Dl3, Dl4 Dl2, Dl6
(L+So)	8	Ml mi me	L1, L4, So1 L2 So3–6

**Table 2b.** Chaetotaxy of *Endonura paracentaurea* sp. n.: chaetotaxy of antennae.

Segment, Group	Number of chaetae	Segment, Group	Number of chaetae adult
I	7	IV ap	or, 8 S, i, 12 mou, 6 brs, 2 iv
II	11		
III ve	5 sensilla AO III		
	5		8 bs, 5 miA
vc	4	ca	2 bs, 3 miA
vi	4	cm	3 bs, 1 miA
d	5	cp	8 miA, 1 brs

**Table 2c.** Chaetotaxy of *Endonura paracentaurea* sp. n.: postcephalic chaetotaxy.

	Terga				Legs				
	Di	De	Dl	L	Scx2	Cx	Tr	Fe	T
Th I	1	2	1	-	0	3	6	13	19
Th II	3	2+s	3+s+ms	3	2	7	6	12	19
Th III	3	3+s	3+s	3	2	8	6	11	18
							Sterna		
Abd I	2	3+s	2	3	VT: 4				
Abd II	2	3+s	2	3	Ve: 5–6; chaeta Ve1 present				
Abd III	2	3+s	2	3	Vel:4–5; Fu: 4–5 me, 4–5 mi				
Abd IV	2	2+s	3	6	Vel: 4; Vec: 2; Vei: 2; Vl: 4				
Abd V	(3+3)	5+s			Ag: 3; Vl: 1, L‘: 1				
Abd VI		7			Ve: 13–14; An: 2mi				

#### Etymology.

The name “*paracentaurea*” refers to a strong similarity of the new species to *E. centaurea*.

#### Diagnosis.

Habitus typical of the genus *Endonura*. Dorsal tubercles present and well developed. 2+2 pigmented eyes. Buccal cone short, labrum nonogival. Head with chaetae A, B, C and D. Chaetae O and E absent. Tubercles Cl and Af separate. Tubercles Dl and (L+So) on head with six and eight chaetae respectively. Tubercles Di on Th I present. Tubercles De on Th II and III with three and four chaetae respectively. Tubercles L on Abd III and IV with three and six chaetae respectively. Abd IV and V with eight and three tubercles respectively. Claw without inner tooth. Tibiotarsi with chaetae B4 and B5 short.

#### Description.

General. Body length (without antennae): 0.90 to 1.65 mm (holotype: 1.65 mm). Colour of the body white. 2+2 small black eyes, in a typical arrangement for the genus (Fig. 13).

*Chaetal morphology*. Dorsal ordinary chaetae of five types: long macrochaetae, short macrochaetae, very short macrochaetae , mesochaetae and microchaetae. Long macrochaetae thick, slightly arc-like or straight, narrowly sheathed, feebly serrated, apically rounded or pointed (Figs 13, 15, 17). Macrochaetae Mc and Mcc morphologically similar to long macrochaetae, but shorter. Mesochaetae similar to ventral chaetae, thin, smooth and pointed. Microchaetae similar to mesochaetae, but apparently short. S–chaetae of tergites thin, smooth and short, notably shorter than nearby macrochaetae (Figs 13, 15, 18).

*Antennae*. Typical of the genus. Dorsal chaetotaxy of Ant III–IV as in Fig. 11 and Table 2b. S–chaetae of Ant IV of medium length and moderately thickened, S4 and S7 notably longer than other S–chaetae, S1 distinctly shorter and thinner than others (Fig. 11). Apical vesicle poorly developed, unilobate. Ventral chaetotaxy of Ant III–IV as in Fig. 12 and Table 2b.

*Mouthparts*. Buccal short with labral sclerifications nonogival. Labral chaetotaxy: 0/2, 4 (Fig. 19). Labium with four basal, three distal and four lateral chaetae, papillae x absent. Maxilla styliform, mandible thin and tridentate.

Dorsal chaetotaxy and tubercles. Head without chaetae E, O, So2 and L3 (Fig. 13). Tubercles Di on Th I differentiated, not fused with tubercles De (Fig. 13). Thorax and abdomen without free chaetae De2 and De3 (Figs 13–15). On Abd I–III, the line of chaetae De1–chaeta s perpendicular to the dorsomedian line. On Abd I-III chaetae De1 notably short (Fig. 15). Cryptopygy absent, Abd VI visible from above.

*Ventral chaetotaxy.* On head, groups Vea, Vem and Vep with 3, 4, 4 chaetae respectively. Group Vi on head with 6 chaetae. On Abd IV, furca rudimentary with 4-5 microchaetae without visible chaetopores (Fig. 16). On Abd V, chaetae Vl and L’ present.

*Legs*. Chaetotaxy of legs as in Table 1a. Claw without internal tooth. On tibiotarsi, chaeta M present and chaetae B4 and B5 relatively short and pointed.

#### Remarks.

Morphologically, *E. paracentaurea* sp. n. strongly recalls *E. centaurea* Cassagnau et Péja, 1979, a form shortly described from Greece (Cassagnau and Péja 1979) and recently redescribed (Smolis 2016). Both species are characterised in having white body, same number of chaetae on tubercles Dl and Af on head, same number of dorsal chaetae on Th and Abd and microchaetae on furca rudimentary. Nevertheless, the new species can be recognised by presence of pigment on eyes (in *centaurea* absent), presence of eight chaetae (L+So) on head (in *centaurea* nine), absence of non-reticulate area within tubercle Af on head (in *centaurea* present) and presence of tubercle Di on Th I (in *centaurea* absent).

### Endonura turkmenica sp. n.

Taxon classificationAnimaliaCollembolaNeanuridae

http://zoobank.org/AC92D847-D212-4D5D-9F9C-F81D24AFF82C

Figs 20–34 , Table 3a

#### Type material.

Holotype: male on slide, Turkmenistan, south-western part of the country (Balkan velayat), Magtymguly (previously Kara-Kala) Area, foothills of the southern slope of the Kopet Dag mountain range, near village Juvankala, right tributary of Sumbar river, leaf litter under elm *Ulmus* spp, 4.II.1977, leg. A. Babenko, A. Uvarov, T. Zheltikova (MSPU). Paratype: juvenile on slide, same data as holotype (DIBEC).

**Figures 20–34.**
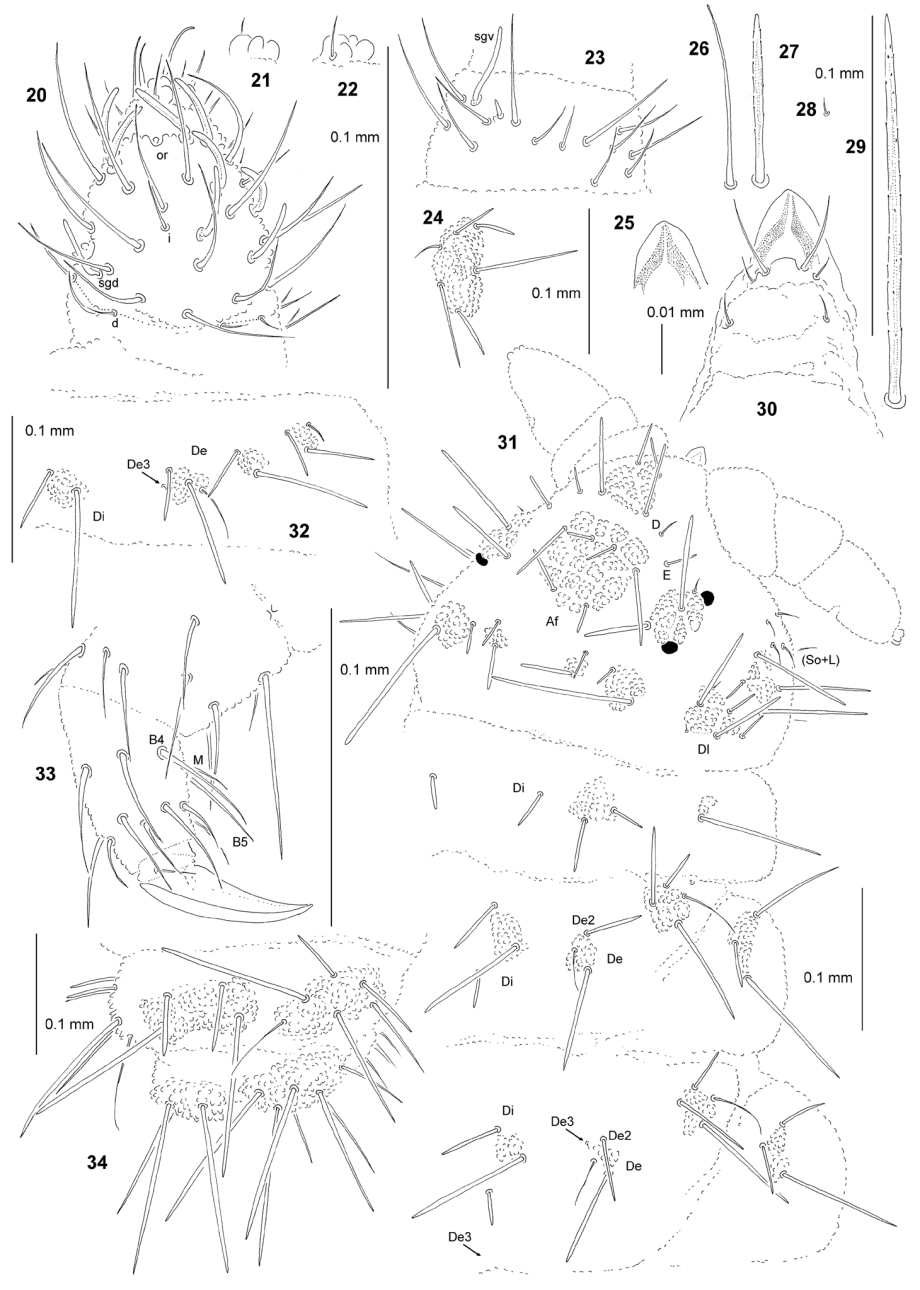
*Endonura turkmenica* sp. n.: **20** dorsal chaetotaxy of Ant III–IV **21** apical bulb, ventral view **22** apical bulb, dorsal view **23** ventral chaetotaxy of Ant III **24** tubercle L of Abd IV **25** ventral sclerifications of labrum **26** sensillum of Abd V **27** chaeta Di2 of Abd V **28** chaeta Di3 of Abd V **29** chaeta Di1 of Abd V **30** chaetotaxy of labrum **31** chaetotaxy of head and Th (holotype), dorsolateral view **32** chaetotaxy of Abd II **33** leg II, chaetotaxy of T and Fe, lateral view 34 chaetotaxy of Abd V–VI, dorsolateral view.

**Table 3a.** Chaetotaxy of *Endonura turkmenica* sp. n.: cephalic chaetotaxy dorsal side.

Tubercle	Number of chaetae	Types of chaetae	Names of chaetae
Cl	4	Ml Mc	F G
Af	10	Ml Mc Mcc	B A, E C, D
Oc	3	Ml Mc mi	Ocm Ocp Oca
Di	2	Mc Mcc	Di1 Di2
De	2	Ml Mcc	De1 De2
Dl	6	Ml Mcc mi	Dl5, Dl1 Dl3, Dl4 Dl2, Dl6
(L+So)	8	Ml Mcc me	L1, L4, So1 L2 So3–6

**Table 3b.** Chaetotaxy of *Endonura turkmenica* sp. n.: chaetotaxy of antennae.

Segment, Group	Number of chaetae	Segment, Group	Number of chaetae adult
I	7	IV ap	or, 8 S, i, 12 mou, 6 brs, 2 iv
II	12		
III ve	5 sensilla AO III		
	5		8 bs, 5 miA
vc	4	ca	2 bs, 3 miA
vi	4	cm	3 bs, 1 miA
d	5	cp	8 miA, 1 brs

**Table 3c.** Chaetotaxy of *Endonura turkmenica* sp. n.: postcephalic chaetotaxy.

	Terga				Legs				
	Di	De	Dl	L	Scx2	Cx	Tr	Fe	T
Th I	1	2	1	-	0	3	6	13	19
Th II	3	2+s	3+s+ms	3	2	7	6	12	19
Th III	3	3+s	3+s	3	2	8	6	11	18
							Sterna		
Abd I	2	3+s	2	3	VT: 4				
Abd II	2	3+s	2	3	Ve: 6; chaeta Ve1 present				
Abd III	2	3+s	2	3	Vel: 5; Fu: 7 me, 0 mi				
Abd IV	2	2+s	3	6	Vel: 4; Vec: 2; Vei: 2; Vl: 4				
Abd V	(3+3)	5+s			Ag: 3; Vl: 1, L’: 1				
Abd VI		7			Ve: 14; An: 2mi				

#### Etymology.

The name *turkmenica* is derived from Turkmenistan, the name of the country where the species was found.

#### Diagnosis.

Habitus typical of the genus *Endonura*. Dorsal tubercles present and well developed. 2+2 pigmented eyes. Buccal cone relatively long, labrum ogival. Head with chaetae A, B, C, D and E. Chaeta O absent. Tubercles Cl and Af separate. Tubercles Dl and (L+So) on head with six and eight chaetae respectively. Tubercles Di on Th I absent. Tubercles De on Th II and III with three and four chaetae respectively. Tubercles L on Abd III and IV with three and six chaetae respectively. Abd IV and V with eight and three tubercles respectively. Claw without inner tooth. Tibiotarsi with chaetae B4 and B5medium size.

#### Description.

General. Body length (without antennae): 0.50 (juvenile) to 1.45 mm (holotype: 1.45 mm). Colour of the body bluish-grey. 2+2 large black eyes, in a typical arrangement for the genus (Fig. 31).

*Chaetal morphology*. Dorsal ordinary chaetae of five types: long macrochaetae, short macrochaetae, very short macrochaetae, mesochaetae and microchaetae. Long macrochaetae relatively thin, straight, narrowly sheathed, serrated, apically pointed (Fig. 29). Macrochaetae Mc and Mcc morphologically similar to long macrochaetae, but much shorter (Fig. 27). Mesochaetae similar to ventral chaetae, thin, smooth and pointed. Microchaetae similar to mesochaetae, but apparently short (Fig. 28). S–chaetae of tergites thin, smooth and short, shorter than nearby macrochaetae (Figs 26, 31, 32, 34).

*Antennae*. Typical of the genus. Dorsal chaetotaxy of Ant III–IV as in Fig. 20 and Table 3b. S–chaetae of Ant IV of medium length and thickened, S5 and S6 slightly longer and thicker than others. Apical vesicle distinct, multilobate (Figs 21, 22). Ventral chaetotaxy of Ant III–IV as in Fig. 23 and Table 3b.

*Mouthparts*. Buccal cone long with labral sclerifications ogival (Fig. 25). Labrum chaetotaxy: 0/2, 4 (Fig. 30). Labium with four basal, three distal and four lateral chaetae, papillae x absent. Maxilla styliform, mandible thin and tridentate.

*Dorsal chaetotaxy and tubercles*. Head without chaetae O, So2 and L3 absent (Fig. 31). Tubercles Di on Th I not differentiated (Fig. 31). On Th and Abd, chaetae De2 and De3 not free (Figs 31, 32), chaetae De3 notably short and hard to detect. On Abd I–III, the line of chaetae De1–chaeta s non perpendicular to the dorsomedian line. Cryptopygy absent, Abd VI visible from above.

*Ventral chaetotaxy*. On head, groups Vea, Vem and Vep with 3, 4, 4 chaetae respectively. Group Vi on head with six chaetae. On Abd IV, furca rudimentary without microchaetae. On Abd IV, group L without free chaeta (Fig. 24). On Abd V, chaetae Vl and L’ present.

*Legs*. Chaetotaxy of legs as in Table 1a. Claw without internal tooth. On tibiotarsi, chaeta M present and chaetae B4 and B5 relatively long and pointed (Fig. 33).

#### Remarks.

*Endonura turkmenica* sp. n. seems to be the closest *E. ceratolabralis* Smolis et al., 2016 recently described from western part of Iran (Smolis et al. 2016a). They differ in a number of characters: maximum length of body without antennae (in *turkmenica* 1,45 mm, in *ceratolabralis* 2,55), location of chaeta E on head (in *turkmenica* free, in *ceratolabralis* connected with tubercle Af), labral formula of chatotaxy (in *turkmenica*: 0/2,4; in *ceratolabralis*: 0/2, 2), number of chaetae (L+So) on head (in *turkmenica* eight, in *ceratolabralis* nine) and presence/absence of tubercle Di on Th I (in *turkmenica* absent, in *ceratolabralis* present). Additionally, chaetae De3 on Th III and Abd I-III are very minute and can be easily overlooked in *turkmenica* (in *ceratolabralis* well visible and slightly shorter than De2).

## Discussion

Unlike most springtails, members of the family Neanuridae completely lack a molar plate on the mandibles and have suctorial mouthparts. Therefore, they are sometimes called sucking forms in contrast to chewing taxa, where mandibles comprise an outlined structure. The highest degree of simplicity of this part of body can be observed within the subfamily Neanurinae, where the majority of genera and species are characterized by a styliform maxilla consisting of no more than one dentate lamella and a thin mandible, usually tridentate with an apical tooth seldom subdivided. For this reason, authors of taxonomic descriptions have usually devoted little attention to the construction of such elements as mandibles or maxillae. Additionally, characteristics of other mouth structures, e.g. the labrum, are frequently omitted from most papers. Interestingly, observations on the genus *Endonura* and other Neanurinae show that the apical part of the labrum can be modified, and its shape is constant and characteristic of the species. In the light of these facts, this mouthpart element is very useful and should be added to the list of diagnostic features in the Neanurinae taxonomy.

Up to date, two main types of shapes of the apical part of the labrum have been observed within *Endonura* and other Neanurinae: nonogival and ogival (Deharveng and Weiner 1984). The latter type, present in two species described herein: *E. longirostris* sp. n. and *E. turkmenica* sp. n., is characterized by a strongly tapered and sharp end. What is interesting and noteworthy for this type seems to be generally less widespread but appears to be more common in representatives of the genus living at lower geographic latitudes. It is important to question the reasons for such modifications of the labrum and the causes of such a strong elongation of this structure in some species. It is worth noting that such elongation of the labrum is rather not observed in taxa with the first nonogival type.

Recent observations and studies on the diet of some Neanurinae species probably provide an answer to the above question (Smolis 2009, Hoskins et al. 2015). Apparently, an important, and perhaps primary, food resource for this subfamily are slime molds. Like most Neanurinae, this group of organisms can be found mainly in forest habitats, moist and rich in organic matter, in particular in different kinds of decaying wood. It is highly probable that the observed differences in the shape of the labrum allow some Neanurinae species to feed on certain species of slime molds and, in consequence, to reduce interspecies competition. Undoubtedly, to clarify this intricate aspect of the morphology and ecology of Neanurinae, further studies on the *Endonura* species with different modifications of the labrum, are needed.

## Supplementary Material

XML Treatment for Endonura longirostris

XML Treatment for Endonura paracentaurea

XML Treatment for Endonura turkmenica
